# On the plasticity of amyloid formation: The impact of destabilizing small to large substitutions on islet amyloid polypeptide amyloid formation

**DOI:** 10.1002/pro.4539

**Published:** 2023-02-01

**Authors:** Lakshan Manathunga, Rehana Akter, Alexander Zhyvoloup, Carlos Simmerling, Daniel P. Raleigh

**Affiliations:** ^1^ Department of Chemistry Stony Brook University Stony Brook New York USA; ^2^ Laufer Center for Physical and Quantitative Biology, Stony Brook University Stony Brook New York USA; ^3^ Research Department of Structural and Molecular Biology University College London London UK

**Keywords:** amylin, amyloid, IAPP, kinetics, steric zippers

## Abstract

Amyloids are partially ordered, proteinaceous, β‐sheet rich deposits that have been implicated in a wide range of diseases. An even larger set of proteins that do not normally form amyloid in vivo can be induced to do so in vitro. A growing number of structures of amyloid fibrils have been reported and a common feature is the presence of a tightly packed core region in which adjacent monomers pack together in extremely tight interfaces, often referred to as steric zippers. A second common feature of many amyloid fibrils is their polymorphous nature. We examine the consequences of disrupting the tight packing in amyloid fibrils on the kinetics of their formation using the 37 residue polypeptide hormone islet amyloid polypeptide (IAPP, amylin) as a model system. IAPP forms islet amyloid in vivo and is aggressively amyloidogenic in vitro. Six Cryo‐EM structures of IAPP amyloid fibrils are available and in all Gly24 is in the core of the structured region and makes tight contacts with other residues. Calculations using the ff14SBonlysc forcefield in Amber20 show that substitutions with larger amino acids significantly disrupt close packing and are predicted to destabilize the various fibril structures. However, Gly to 2‐amino butyric acid (2‐carbon side chain) and Gly to Leu substitutions actually enhance the rate of amyloid formation. A Pro substitution slows, but does not prevent amyloid formation.

## 1 INTRODUCTION

**FIGURE 1:**
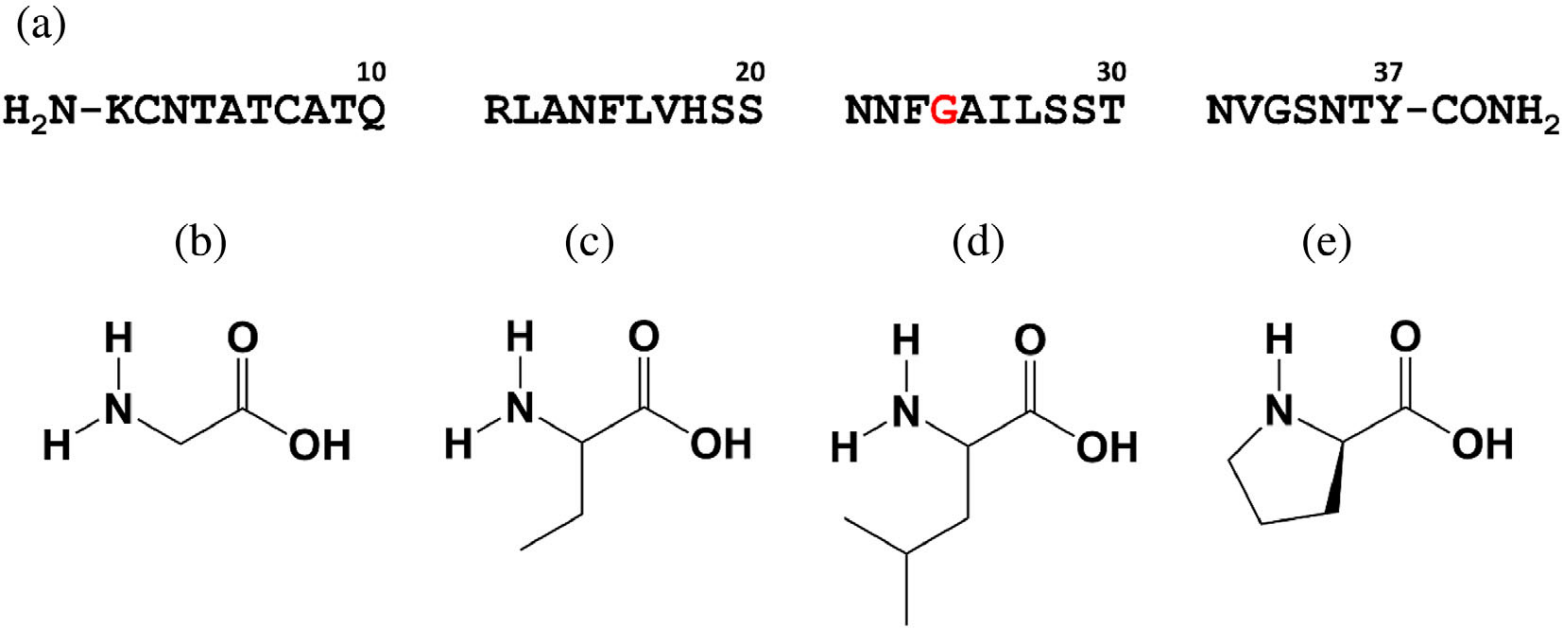
(a) Sequence of wild type human islet amyloid polypeptide (hIAPP) where the 24th residue is a (b) glycine. Three hIAPP variants were studied in which the 24^th^ residue was replaced with (c) 2‐amino butyric acid, (d) leucine, or (e) proline

Amyloids are partially ordered, proteinaceous, β‐sheet rich deposits that have been implicated in a wide range of diseases (Rambaran & Serpell, 2008). An even larger set of proteins that do not normally form amyloid in vivo can be induced to do so in vitro (Chiti & Dobson, 2017; Eisenberg & Jucker, 2012). A growing number of structures of amyloid fibrils have been reported and a common feature is the presence of closely packed segments of the protein chain where adjacent monomers pack together in close proximity in a tight interface (Riek, 2017). A second feature of many amyloid fibrils is their polymorphic nature; seemingly small changes in solution conditions or mutations can lead to different fibril structures (Eisenberg & Jucker, 2012; Fändrich et al., 2018; Meinhardt et al., 2009; Qiang et al., 2017). We examine the consequences of disrupting tight packing in amyloid fibrils on the kinetics of their formation using the 37 residue polypeptide hormone islet amyloid polypeptide (IAPP, also known as amylin) as a model system (Figure 1).

Human IAPP (hIAPP) is the protein component of pancreatic islet amyloid associated with type‐2 diabetes (Figure 1) (Abedini et al., 2016; Akter et al., 2016; Westermark et al., 2011). It is one of the most amyloidogenic natural sequences known and forms amyloid even more rapidly than the Aβ peptide of Alzheimer's disease. The sequence of IAPP is highly conserved, however not all species develop type‐2 diabetes and those which do not produce and secrete a form of IAPP that is non amyloidogenic in vivo (Noh et al., 2020; Westermark et al., 1990). In contrast, species that develop type‐2 diabetes also develop islet amyloid and produce an amyloidogenic version of IAPP. Of historical interest is the observation that rat/mouse IAPP is not amyloidogenic under normal circumstances in‐vitro, and neither rats nor mice suffer from type‐2 diabetes (Cao et al., 2010). The rodent and human sequences differ at 6 positions, 5 of which are between residues 20 and 29. These observations focused early attention on the 20–29 region of IAPP as a segment critical for modulating amyloidogenicity. Subsequent work has shown that substitutions outside this region can also influence amyloid formation, but the segment is clearly important (Abedini & Raleigh, 2006). A number of peptides derived from this region which contain the _22_NFGAIL_27_ sequence have been shown to form amyloid in isolation (Moriarty & Raleigh, 1999; Westermark et al., 1990).

Gly24 is conserved in all known IAPP sequences, perhaps to facilitate receptor binding as it is in the so called “by pass” region of the chain which is believed to be important for receptor binding (Bower et al., 2018; Cao et al., 2022). Here we examine the consequences of small to large substitutions of residue 24 on hIAPP amyloid formation (Figure 1). These substitutions are designed to disrupt the tight packing in the various hIAPP amyloid fibril structures. Energy decomposition using the ff14SBonlysc (Nguyen et al., 2014) forcefield indicates that small to large substitutions at this site are predicted to lead to unfavorable steric clashes that destabilize fibrils. However, the substitutions do not inhibit amyloid formation, rather they accelerate it. We also examine the consequences of a Proline replacement at position 24. Proline is well known to be unfavorable in β‐sheet. However, the G24P substitution slows, but does not prevent amyloid formation.

## 2 RESULTS

### 2.1 Analysis of the environment of Gly24 in known structures of hIAPP amyloid fibrils

**FIGURE 2:**
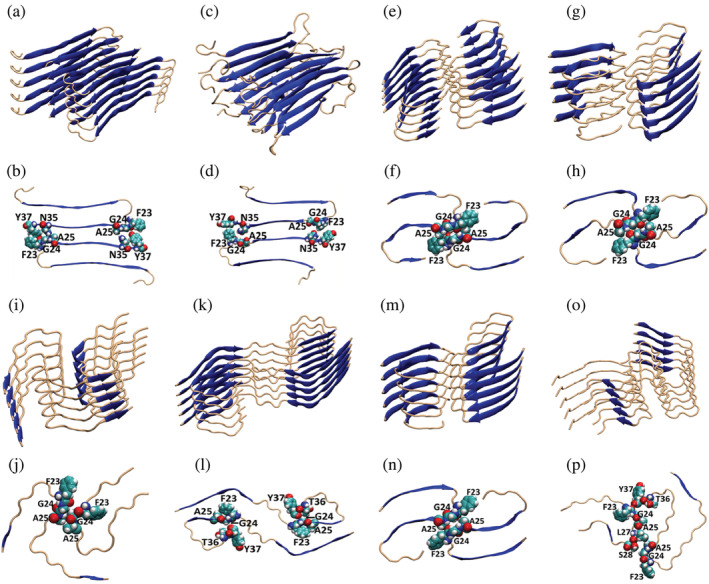
Location of G24 and its surrounding residues in different structural models of the human islet amyloid polypeptide (hIAPP) amyloid fibril. Five layered ribbon diagrams of the different fibril structures are illustrated for each of the hIAPP fibril models and a top down view of a cross section for each model is illustrated immediately below. (a, b) Model developed based on crystallographic studies of small steric zipper peptides (coordinates kindly provided by the authors); (c, d) model developed by solid state NMR derived constrains (coordinates kindly provided by the authors); (e, f) (pdb = 6Y1A) model developed by Cryo‐EM studies of synthetic IAPP conducted at pH 6.0; (g, h) (pdb = 6ZRF) model developed using Cryo‐EM studies of synthetic IAPP conducted at pH 6.8; (i–p) (pdb codes, 7M61, 7M62, 7M64, 7M65 respectively from left to right) different polymorphic models derived from Cryo‐EM studies of hIAPP seeded by human tissue extracts of islet amyloid. For the Cryo‐EM models (e, g, i, m) residues 13–37 and for (k) and (o) residues 6–37 were reported as ordered. For model (o), inequivalent chains were reported with residues 6–37 for one chain and residues 14–36 of the second

Six cryo‐electron microscopy (Cryo‐EM) structures of fibrils formed by wild type hIAPP have been reported (Cao et al., 2021; Gallardo et al., 2020; Röder et al., 2020). In addition, a model based on crystal structures of short hIAPP derived peptides has been developed, as has another model based on solid state NMR studies of intact hIAPP (Luca et al., 2007; Wiltzius et al., 2008). The various models differ in their details but have some common features (Figure 2). They all contain well‐ordered intermolecular β‐sheets. Gly24 has limited solvent exposure in all of the models except for the solid state NMR based model (Figure 2c,d) and one of the Cryo‐EM based models (Figure 2o,p, Table 1). Even in those two cases the solvent exposure is less than 40%. Gly24 is located in an ordered loop region in all the models except for some of the chains in the solid state NMR model (Figure 2c,d) and one of inequivalent monomer stacks in one of the Cryo‐EM models (Figure 2i,j). Even though it is in a loop like structure, Gly24 is in the core of the amyloid structures in all models. In contrast the N‐terminal first 8–10 residues are not defined in most of the structures. In four of the Cryo‐EM models (Figure 2e–j,m,n) the contact between Gly24 of one chain and Gly24 of the other chain in the same layer is the closest distance between the backbone of the two chains. In three of the models (Figure 2a–d,k,l) Gly24 in one chain is in close proximity to the C‐terminus of the other monomer in the same layer. In the Cryo‐EM model which contains inequivalent chains in each layer, one Gly24 is in close proximity to the C‐terminus of the other chain and the other Gly24 is in close proximity to Ser28 (Figure 2o,p). Thus, Gly24 makes close contacts in all reported structural models of the hIAPP fibril. We also examined the backbone conformations of Gly24 in each of the models. Glycines sometimes adopt conformations with a positive value of the backbone dihedral angle *φ* and such a conformation is energetically unfavorable for the other 19 genetically coded amino acids. In all of the structures with the exception of one of the Cryo‐EM based models (PDB code 7M65; Figure 2o,p) Gly24 adopts a structure with a negative *φ* (Table 1 and Figure S1).

**TABLE 1:** Total Van der Waal's (VDW) energy of the side chain of the 24th residue calculated after minimizing the fibril structures of hIAPP, G24 2Abu‐hIAPP, G24L‐hIAPP, and G24P‐hIAPP for 10,000 steps in Amber 20

Fibril structure	Exp. method	Side chain VDW energy (kcal mol^−1^)				Psi angle (°)	Phi angle (°)	% Fractional SASA of G24
		hIAPP	G24 2Abu‐hIAPP	G24L‐hIAPP	G24P‐hIAPP			
	X‐ray crystallography	−1.1	29.8	17.7	^a^	−120.2	−112.8	0.0
Stack A	Solid state NMR	−0.8	239.9	^a^	27.9	116.6	−56.1	17.9
Stack B	Solid state NMR	−0.5	42.6	^a^	41.3	150.3	−168.6	38.1
6Y1A	Cryo‐EM	−3.4	20.1	^a^	^a^	167.4	−88.3	0
6ZRF	Cryo‐EM	−0.9	13.4	44.9	26.1	−177.1	−169.5	0
7M61—Stack A	Cryo‐EM	−1.3	^a^	^a^	−3.2	172.3	−127.3	1.35
7M61—Stack B	Cryo‐EM	−0.9	^a^	^a^	2.0	−63.0	−79.6	0.0
7M62	Cryo‐EM	−1.1	−1.4	0.0	24.2	123.0	−126.5	0.9
7M64	Cryo‐EM	−1.1	25.9	^a^	17.2	169.0	−75.5	0.0
7M65—Stack A	Cryo‐EM	−0.5	13.7	78.1	33.8	−121.8	149.1	21.8
7M65—Stack B	Cryo‐EM	−1.0	−0.8	5.0	24.0	162.5	173.5	11.9

*Note*: If the two stacks of a fibril structure are inequivalent, the VDW energies for both stacks are listed. Psi and phi angles of the 24th residue in all the fibril structures were obtained using visual molecular dynamics (VMD). Percent fractional solvent accessible surface area (SASA) calculated for the 24th residue (Gly) of hIAPP fibril structures, calculated relative to a tripeptide (Phe, Gly, Ala) in an extended conformation where *ɸ* = 180° and Ψ = 180°. SASA values were calculated using the measure SASA command in VMD. Cooridinates for the solid state NMR and X‐ray crystalloraphy based structures were supplied.

^a^ High energy (greater than 999,999.999 kcal mol^−1^).

### 2.2 Energy decomposition indicates that Gly24 to 2‐Amino butyric acid, Gly24 to Leu and Gly24 to Pro substitutions destabilize hIAPP amyloid fibrils

We used energy decomposition to examine the consequences of replacing Gly24 with a 2 carbon sidechain (2‐amino butyric acid) and with Leu (Figure 1). We used the method of steepest descent to minimize the structures and then calculated the Van der Waal's energy for Gly, 2‐Abu and a Leu at residue 24 using the Amber ff14SBonlysc forcefield. The Leu substitution is predicted to lead to significant destabilizing steric clashes in all structures with the exception of one chain of one of the Cryo‐EM based models (Tables 1 and S1, Figure 2k,l; PDB code 7M62). The predicted destabilization is large in all of the other structures, and several were predicted to be destabilized by more than 40 kcal mol^−1^. The effects of 2‐Abu are less drastic, but are still predicted to be significantly destabilizing for at least one of the two stacks in seven of the eight structural models. This analysis confirms the conclusions from the visual inspection and indicates that the substitutions are expected to be destabilizing in the context of known hIAPP fibril structures, particularly for the Leu replacement. Energy decomposition shows that the proline replacement also leads to significant predicted steric clashes in the fibril structure, with the exception one chain of one Cryo‐EM structure (7M61).

### 2.3 Gly24 to 2‐Abu and Gly24 to Leu substitutions accelerate, rather than inhibit amyloid formation

We next examined the consequences of the substitutions on amyloid formation using thioflavin‐T assays and transmission electron microscopy (TEM). Thioflavin‐T is a small dye that is widely used in studies of amyloid formation, including for numerous studies of IAPP amyloid formation (Abedini et al., 2016; Noh et al., 2020). The fluorescence quantum yield of thioflavin‐T increases significantly when bound to the cross β structure of amyloid fibrils. The dye is an extrinsic probe of amyloid formation, but has been shown not to perturb hIAPP amyloid formation under the conditions of our experiments. None the less, we also recorded TEM images at the end of each kinetic experiment to confirm the presence of amyloid fibrils. This is important as at least one variant of IAPP has been reported to give false negatives in thioflavin‐T assays (Wong et al., 2016).

**FIGURE 3:**
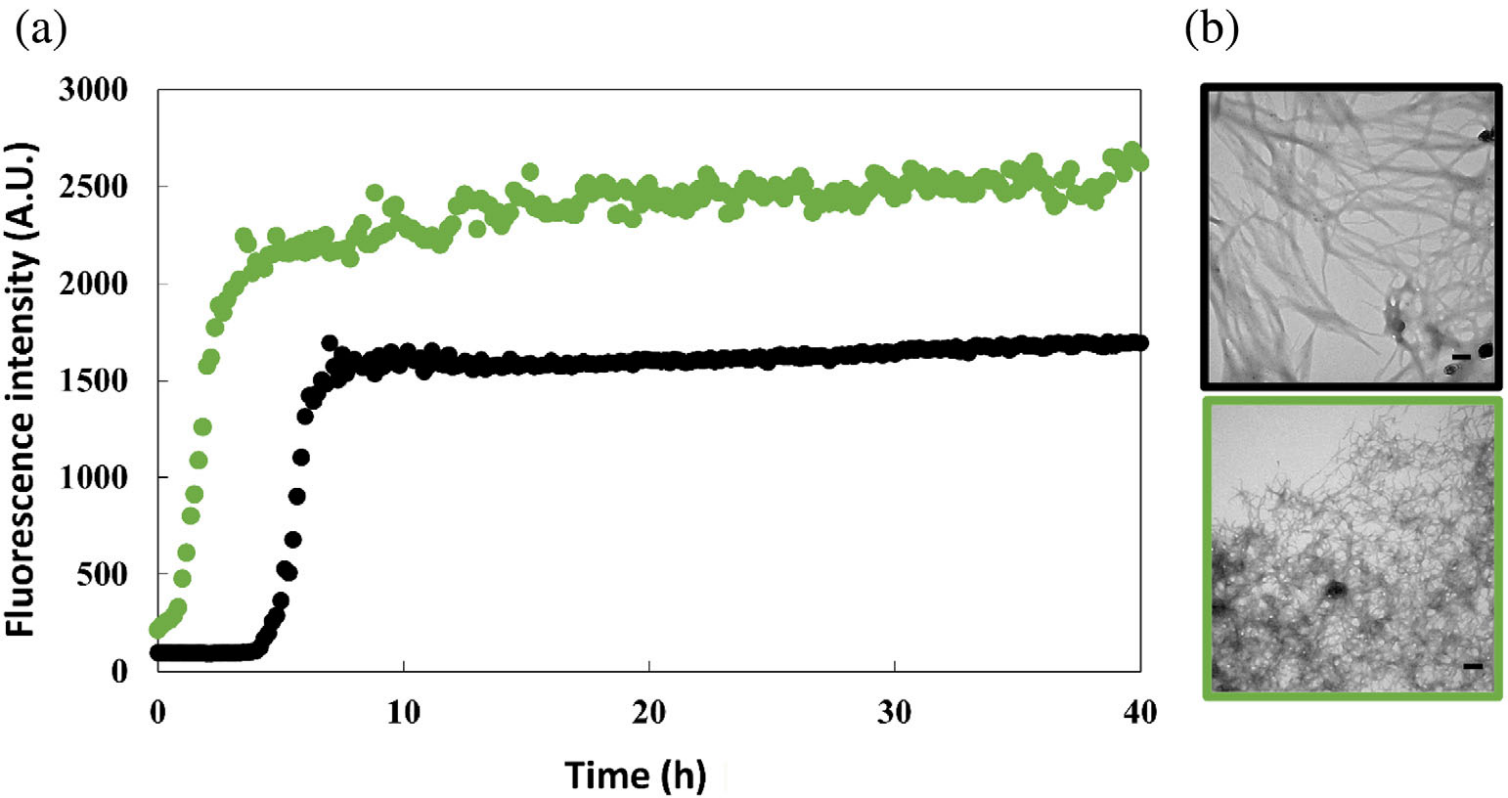
(a) Amyloid formation kinetics of human islet amyloid polypeptide (hIAPP) (black) and G24L‐hIAPP (green) measured using the fluorescence of thioflavin‐T in 10 mM phosphate buffered saline (140 mM KCl), pH 7.4 at 25°C. (b) Transmission electron microscopy images of amyloid fibrils of hIAPP (black) and G24L‐hIAPP (green), taken after the kinetic experiments in (a). Scale bars represent 100 nm

**FIGURE 4:**
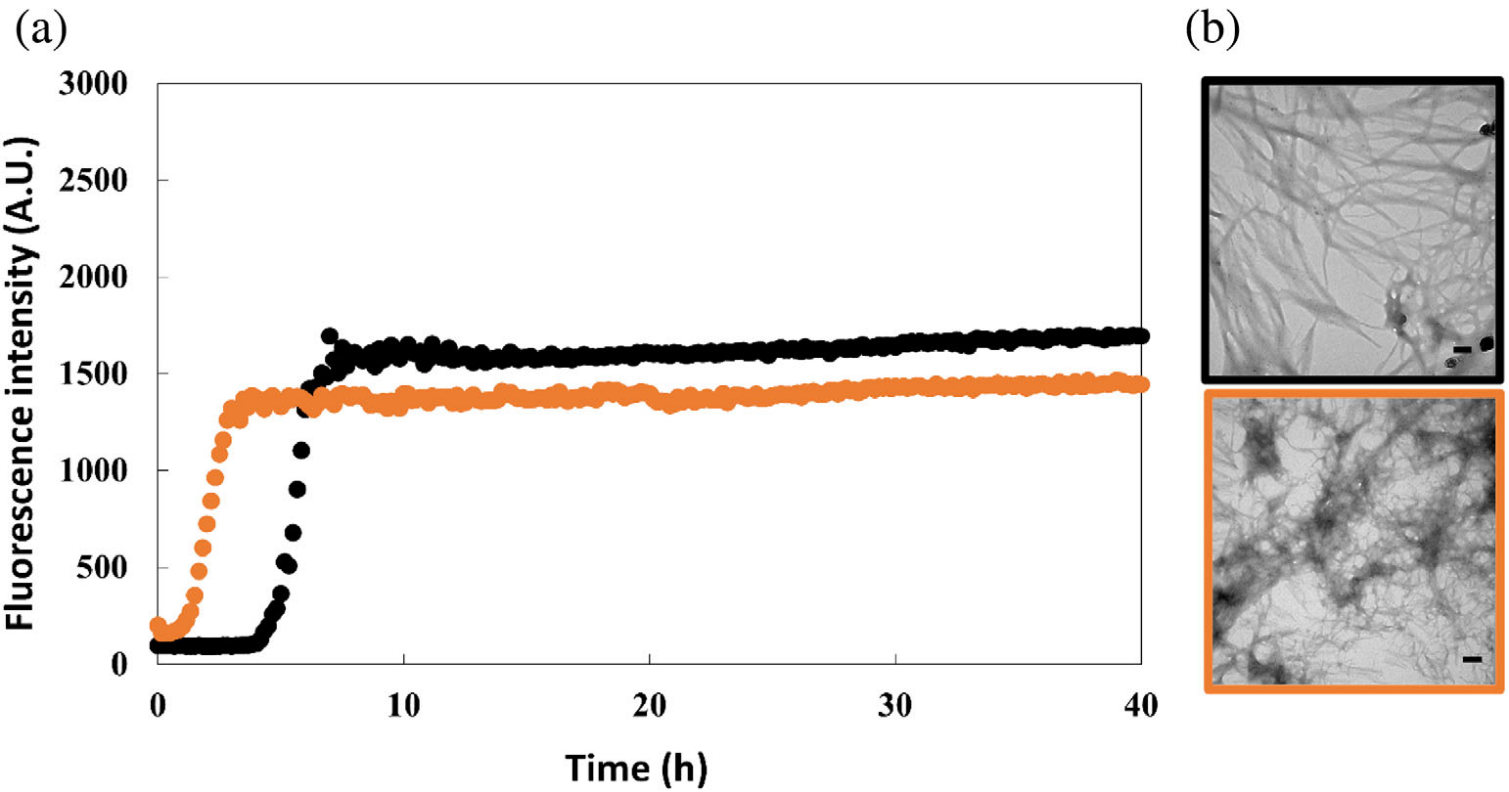
(a) Amyloid formation kinetics of human islet amyloid polypeptide (hIAPP) (black) and G24 2Abu‐hIAPP (orange) measured using the fluorescence of thioflavin‐T in 10 mM phosphate buffered saline (140 mM KCl), pH 7.4 and in 25°C. (b) Transmission electron microscopy images of amyloid fibrils of hIAPP (black) and G24 2Abu‐hIAPP (orange), taken after the kinetic experiments in (a). Scale bars represent 100 nm. The kinetic curve for hIAPP and the transmission electron microscopy (TEM) image of the hIAPP fibrils are identical to the ones shown in Figure 3

The thioflavin‐T curves for wild type and the two variants each have the expected sigmoidal shape for a hIAPP thioflavin‐T assay. Amyloid formation by both variants is faster than amyloid formation by wild type hIAPP in phosphate buffered saline (PBS), (Figures 3, 4, and S2). T_50_, the time required to reach 50% of the signal change in a thioflavin‐T assay is widely used as a parameter to describe the time to form amyloid. The T_50_ for hIAPP is 5.78 (±0.79) hours under the conditions of our experiments, while the T_50_ value for G24L‐hIAPP is 1.95 (±0.05) hours and the value for G24 2Abu‐hIAPP is 2.07 (±0.08) hours. Aliquots were removed at the end of each kinetic experiment and analyzed by TEM (Figures 3 and 4). The TEM images reveal typical matts of fibrils for all three peptides.

We tested the ability of both G24 2‐Abu and G24L variants to seed amyloid formation by wild type hIAPP. Seeding refers to the effect of adding a small amount of pre‐formed amyloid fibrils (the “seed”) to an unaggregated sample of polypeptide. The seed acts as a template for rapid amyloid formation. In many cases, self‐seeding leads to bypassing of the lag phase. Self‐seeding refers to the seeds being formed from the same polypeptide. Amyloid fibrils formed by mutants or by different proteins may or may not be effective seeds. Lack of seeding is usually interpreted as indicating that the soluble peptide is not able to readily adopt the structure of the monomers in the seed. In contrast, highly efficient seeding indicates that the monomeric peptide can easily adapt a structure consistent with the structure of the seeds. Thus, a seeding experiment provides indirect evidence for the compatibility of a polypeptide with a specific amyloid structure. Seeds formed by wild type hIAPP effectively seed amyloid formation by wild type and leads to the elimination of the lag phase (Figure S3). Amyloid fibrils formed by the G24 2‐Abu variant proved to be excellent seeds for wild type hIAPP, but the seeds derived from G24L‐hIAPP amyloid fibrils were ineffective at promoting amyloid formation. The data indicates the wild type hIAPP is readily able to adopt the structure of G24 2Abu‐hIAPP amyloid fibrils. In contrast the wild type is much less compatible with the G24L‐hIAPP amyloid fibrils. This suggests that the fibrils formed by G24 2Abu‐hIAPP and G24L‐hIAPP may differ in structure.

### 2.4 A Proline substitution at position 24 slows amyloid formation significantly, but does not prevent it

**FIGURE 5:**
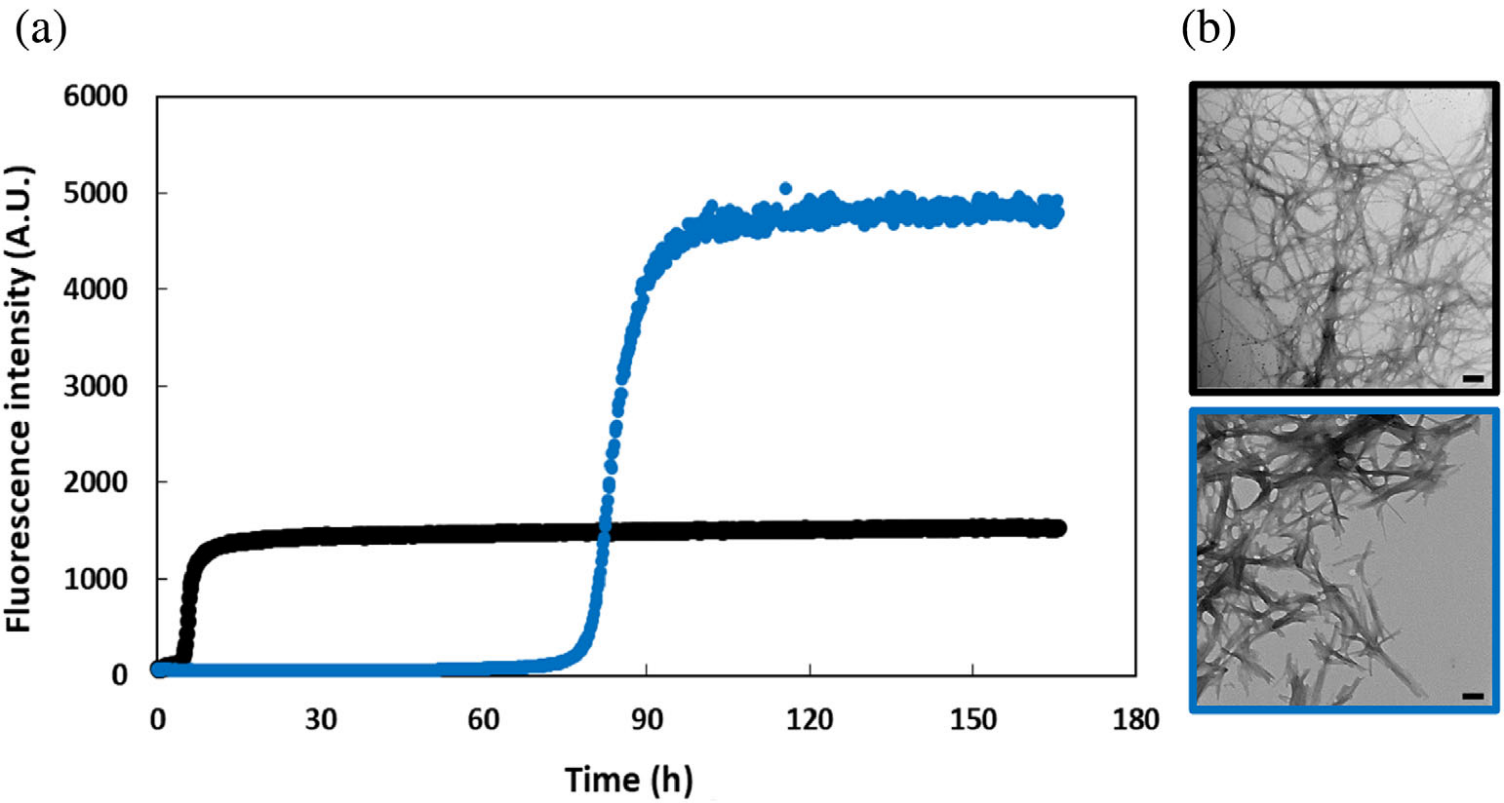
(a) Amyloid formation kinetics of human islet amyloid polypeptide (hIAPP) (black) and G24P‐hIAPP (blue) measured using the fluorescence of thioflavin‐T in 10 mM phosphate buffered saline (140 mM KCl), pH 7.4 and in 25°C. (b) Transmission electron microscopy images of amyloid fibrils of hIAPP (black) and G24P‐hIAPP (blue), taken after the kinetic experiments in (a). Scale bars represent 100 nm. Note the X‐axis is on a different scale compared to Figures 3 and 4

We next examined the consequences of a Proline replacement at position 24. The Proline substitution significantly increased the time to form amyloid relative to wild type hIAPP as judged by the thioflavin‐T assays (Figure 5a). The T_50_ is increased by more than a factor of 15 relative to wild type hIAPP. TEM confirms the presence of amyloid fibrils in the sample (Figure 5b).

### 2.5 The 2‐Abu and Leu substitutions do not significantly alter the solubility of IAPP, but the Pro replacement enhances it

**FIGURE 6:**
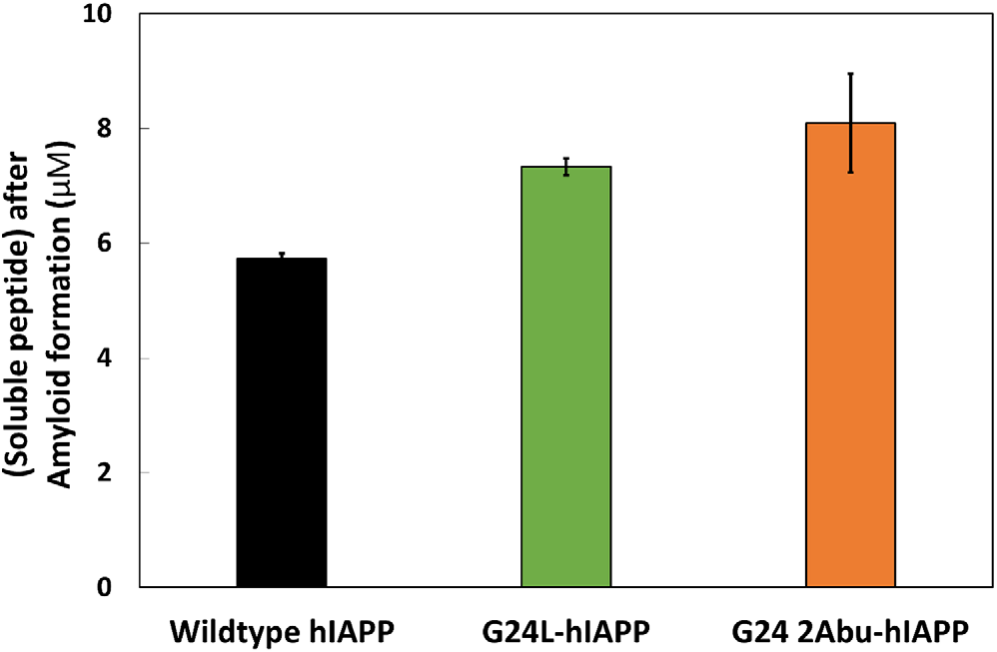
The amount of soluble peptide remaining in solution 5 days after dissolving human islet amyloid polypeptide (hIAPP), G24L‐hIAPP, and G24 2Abu‐hIAPP in 10 mM phosphate buffered saline (140 mM KCl), pH 7.4 and 25°C. The amount of peptide in the supernatant was measured by the micro‐BCA assay after centrifugation (18,000 g for 30 min). None of the differences were judged to be significant (*p* > 0.05 for all the cases)

We next compared the apparent solubility of the Gly24 variants to wild type hIAPP. Samples were dissolved in PBS and allowed to stand for 5 or 7 days (in the case of G24P). These times are sufficient to form amyloid fibrils. Samples were then centrifuged and the concentration of the peptide remaining in solution was determined. Slightly higher concentrations of the G24 2‐Abu and G24L variants were detected compared to wild type, but the small differences are not statistically significant (Figure 6). At first glance it might seem surprising that substitutions which destabilize amyloid fibrils do not lead to an increase in soluble material, however it is important to note that this assay simply measures the peptide remaining in the soluble phase, it does not indicate if the insoluble material is exclusively amyloid fibrils or a mixture of fibrils and less ordered aggregates. Thus, there is no reason to expect a direct one‐to‐one correlation between fibril stability and solubility. The G24P variant behaves differently and lead to a significant increase in apparent solubility, the amount of peptide remaining in the solution was greater than 50 μM.

## 3 DISCUSSION

The combined computational and experimental analysis presented here reveals the plasticity of amyloid formation: substitutions that lead to significant steric clashes in known structures of hIAPP amyloid fibrils did not inhibit amyloid formation, but in the examples studied here accelerated it. Why do the 2‐Abu and Leu substitutions accelerate amyloid formation? They are located within the region which has been proposed to adopt transient β‐sheet structure during in vitro hIAPP amyloid formation (Buchanan Lauren et al., 2013). Rearrangement of this intermediate is thought to lead to a free energy barrier and crossing this barrier is believed to be an important determinant of the lag time. Recent 2D‐IR studies have shown that G24 is a part of the structured region in this intermediate (Dicke et al., 2022). It may be that the larger hydrophobic side chains stabilize the transition state involved in this event. Somewhat similar effects have been observed in protein folding. In that case substitutions which destabilized the hydrophobic core of a globular protein and decreased overall stability lead to a faster folding because they stabilized the transition state for folding via non‐specific hydrophobic interactions (Anil et al., 2005). Another, not mutually exclusive, possibility is that the G24 2‐Abu and G24L substitutions change the conformational landscape of the monomer and lead to higher population of a conformation compatible with the amyloid fibril. Course grained and all atom simulations as well as lattice models indicate that this effect can significantly accelerate amyloid formation and have led to the N* hypothesis (Chakraborty et al., 2020; Li et al., 2010; Nam et al., 2010). The N* hypothesis postulates that amyloid formation initiates from a rarely populated monomer conformation that shares some structural features with the fibril state. The model has successfully accounted for the impact of some mutations on amyloid formation by Aβ and for amyloid formation by the low‐complexity FUS protein (Chakraborty et al., 2020; Kumar et al., 2021). To the best of our knowledge, the approach has not been applied to hIAPP and this could be an interesting avenue for future work. A third possible explanation is provided by the wide range of polymorphs observed for hIAPP amyloid fibrils. It may be that the substitutions lead to formation of a different polymorph that accommodates the small to large substitutions and forms via an alternative pathway compared to wild type hIAPP amyloid fibrils. The inability of the G24L variant to seed amyloid formation by wild type hIAPP is consistent with this scenario. The resolution of TEM is not sufficient to distinguish between potentially different structures; Cryo‐EM studies would be required, but are beyond the scope of the present work. Irrespective of the molecular basis of the effects observed here, the data clearly illustrates the plasticity of the IAPP sequence toward amyloid formation.

## 4 MATERIALS AND METHODS

### 4.1 Peptide synthesis and purification

9‐Flourenylmethylcarbonyl (Fmoc) chemistry on a 0.1 mmol scale with a CEM Liberty Blue peptide synthesizer was used to synthesize human IAPP and its Gly24 variants. Physiologically relevant C‐terminal amidation was provided using Fmoc‐PAL‐PEG‐PS resin (0.19 mmol/eq). The yield was improved using a pseudoproline at position 26 and 27 (Abedini & Raleigh, 2005; Marek et al., 2010). The solution used to cleave the peptide from the resin contained trifluoroacetic acid (92.5%) with, 2.5% 3,6‐Dioxa‐1,8‐octanedithiol, 2.5% triisopropylsilane, and 2.5% water. Cleaved peptides were dissolved in 20% acetic acid (4 mg/ml) and lyophilized for 48 h. Peptides were then dissolved in 100% DMSO (10 mg/ml) to promote disulfide bond formation and incubated at room temperature on a shaker for 5 days, based on the method published by Tam and colleagues (Tam et al., 1991). A 0.45 μm Millex membrane filter was used to filter the material prior to purification via reverse phase high performance liquid chromatography (RP‐HPLC) (Higgins C_18_ 25 mm × 250 mm column). A gradient of water/acetonitrile each containing 0.45% HCl was used for the HPLC purification. The peptide fractions were collected, lyophilized for 48 h before redissolving in 1,1,1,3,3,3‐hexafluoroisopropanol (HFIP), and subjected to a second round of HPLC to remove residual scavengers. The purity of the peptides was confirmed by analytical HPLC and matrix assisted laser desorption ionization time‐of‐flight (MALDI‐TOF) mass spectrometry was used to confirm the mass of the peptides. Human IAPP, expected 3903.6 Da, observed 3903.2 Da; G24L‐hIAPP, expected 3959.4 Da, observed 3958.8 Da; G24 2Abu‐hIAPP, expected 3931.4 Da, observed 3931.7 Da; G24P‐hIAPP, expected 3943.4 Da, observed 3942.8 Da.

### 4.2 Preparation of stock solutions and aliquoted samples

Lyophilized dry peptide was dissolved in HFIP (1 mg/1 ml) and incubated for 4 h at 4°C. A filtration step was done using a 0.22 μm Millex low protein binding Durapore membrane filter. The volume needed from each stock solution to prepare 16 μM aliquots was calculated based on the absorbance (at 280 nm wavelength) of 20 μl aliquots taken from each stock solution. These aliquots were lyophilized and redissolved in 100 μl of 10 mM PBS (140 mM KCl), pH 7.4 and the absorbance was measured. The 16 μM aliquots used for the kinetics experiments were prepared by removing the calculated volumes and lyophilizing for at least 24 h.

### 4.3 Fluorescence assays

Peptide samples were reconstituted in 10 mM phosphate buffer saline (140 mM KCl), pH 7.4, and thioflavin‐T (ThT) was then added. The final peptide concentration was 16 μM and the final ThT concentration was 32 μM. A Corning 96‐well clear bottom plate with a non‐binding coating, was used with a Molecular Devices SpectraMax Gemini EM microplate reader. Fluorescence readings (450 nm excitation and emission at 485 nm) were taken every 10 min at 25°C without any shaking. Seeding experiments were performed by allowing samples of wild type hIAPP, G24 2Abu‐hIAPP, and G24L‐hIAPP to form amyloid fibrils. Seeds from these samples were added to freshly prepared sample of wild type hIAPP. The concentration of seed was 10% (vol/vol).

### 4.4 Transmission electron microscopy

Samples for TEM were prepared at the end of the kinetics assays by blotting 15 μl of material onto Carbon‐coated Formvar 300 mesh copper grids. Fifteen microliters of 1% depleted uranyl acetate (UA) was used to negatively stain each sample. A FEI Bio TwinG^2^ transmission electron microscope at the Central Microscopy Imaging Center at Stony Brook University was used to record images.

### 4.5 Peptide solubility measurements

Dry peptides were dissolved in HFIP to prepare the stock solutions. The concentrations of the stock solutions were measured by using freeze dried aliquots of the stock solutions with the micro‐BCA assay. Based on the stock solution concentrations, aliquots were prepared and lyophilized. The dry peptides were reconstructed in 10 mM phosphate buffer saline (140 mM KCl), pH 7.4 at a final peptide concentration of 200 μM. Immediately after reconstitution 10 μl of the sample was removed and the soluble peptide concentration was measured using the micro‐BCA assay. The remaining peptide samples were covered in parafilm and stored at room temperature for 5 days. After 5 days, a 450 μl portion from each sample were removed and centrifuged at 18,000 g for 30 min (25°C). A 100 μl portion of the centrifuged supernatant was subjected to the micro‐BCA assay to calculate the amount of soluble peptide left after 5 days.

### 4.6 Energy decomposition of hIAPP and Gly24 substituted hIAPP

For hIAPP structures the PDB files were used as starting structures. Missing atoms were added using TLEAP in Amber 20 software package (Case et al., 2018). The structures were prepared for energy minimization using the ff14SBonlysc force field with mbondi3 and GBradii (Nguyen et al., 2013). Energy minimization was done in implicit model GBneck2 for maximum number of 10,000 steps. After energy minimization, energy decomposition was performed using Sander on a per‐residue basis. Then the VDW energy for the sidechain of residue 24 was extracted from the output file. G24L substituted fibril structures were generated by substituting the Gly24 with a Leu residue in Chimera using the Dunbrack rotamer library (Pettersen et al., 2004). After the substitution, the same steps were followed as in hIAPP energy decomposition. To generate G24 2‐Abu substituted fibril structures, Gly24 was first substituted by Ser in Chimera using the Dunbrack rotamer library. Then the Ser side chain was altered into the 2‐amino butyric acid sidechain. The 2‐amino butyric acid, parameters published by Khoury et al. were used (Khoury et al., 2014). After the substitution, the same steps were followed to perform the energy decomposition.

### 4.7 Solvent accessible surface area calculations

Chimera was used to add missing H atoms to all the PDB files of the fibril structures. The solvent accessible surface area (SASA) was calculated using the *measure sasa* command in visual molecular dynamics (Humphrey et al., 1996). All the atoms (sidechain and backbone) of the 24th residue were used for the SASA calculations. Percent SASA values reported are relative to the SASA value of the FGA tripeptide with a capped C‐terminus in an extended conformation (*ϕ*, ψ = 180°, 180°). The tripeptide was generated using TLEAP in Amber20.

## AUTHOR CONTRIBUTIONS

**Lakshan Manathunga:** Conceptualization (equal); data curation (equal); formal analysis (equal); investigation (equal); methodology (equal); software (equal); validation (equal); visualization (equal); writing – original draft (supporting); writing – review and editing (supporting). **Rehana Akter:** Conceptualization (supporting); data curation (equal); formal analysis (supporting); methodology (supporting); validation (equal); writing – review and editing (equal). **Alexander Zhyvoloup:** Conceptualization (supporting); data curation (equal); investigation (supporting); methodology (equal); project administration (supporting); resources (equal); validation (equal). **Carlos Simmerling:** Funding acquisition (supporting); methodology (supporting); project administration (supporting); software (lead); supervision (supporting); validation (equal); writing – review and editing (supporting). **Daniel Raleigh:** Conceptualization (lead); data curation (equal); formal analysis (equal); funding acquisition (lead); investigation (lead); methodology (equal); project administration (equal); supervision (lead); validation (equal); visualization (equal); writing – original draft (lead); writing – review and editing (equal).

## CONFLICT OF INTEREST

The authors declare no competing financial interest.

## Supporting information


**FIGURE S1:** Placement of Φ, Ψ angles of Gly24 in the IAPP fibril structures on a Ramachandran Plot. The favored areas in the Ramachandran plot are displayed in orange and allowed areas are displayed in yellow. If the structure contains symmetric chains the average values are plotted and if the structure contains asymmetric chains the two values are reported separately as Chain A and Chain B
**FIGURE S2:** Triplicates of the thioflavin‐T kinetic assays. Details are provided in the captions to Figure‐3, Figure‐4 and Figure‐5 in the main manuscript. Figures 3, 4 and 5 each display one representative curve for each polypeptide.
**FIGURE S3:** Results of seeding experiments **(A)** amyloid formation kinetics of hIAPP (Black), hIAPP seeded with pre‐formed hIAPP amyloid fibrils (Gray) and hIAPP seeded with pre‐formed G24L‐hIAPP fibrils (green) and **(B)** amyloid formation kinetics of hIAPP (Black), hIAPP seeded with pre‐formed hIAPP amyloid fibrils (Gray) and hIAPP seeded with pre‐formed G24 2Abu‐hIAPP fibrils (yellow) measured using the fluorescence of thioflavin‐T in 10 mM phosphate buffered saline (140 mM KCl), pH 7.4 and in 25°C
**TABLE S1.** Energy decomposition (Total, Side Chain and Backbone energies) of the Van der Waal's energy of the 24^th^ residue calculated after minimizing the fibril structures of hIAPP, G24 2Abu‐hIAPP, G24L‐hIAPP and G24P‐hIAPP for 10000 steps in Amber 20. If the two stacks of a fibril structure are inequivalent energies for both stacks are listed.

## Data Availability

The data that support the findings of this study are available from the corresponding author upon reasonable request.
