# Supplementary material for: On the plasticity of amyloid formation: The impact of destabilizing small to large substitutions on islet amyloid polypeptide amyloid formation
Source: Protein Sci. 2023 Feb 1;32(2):e4539. doi: 10.1002/pro.4539 (PMC9847078; doi:10.1002/pro.4539)
Supplement: Supplementary file 1 — FIGURE S1: Placement of Φ, Ψ angles of Gly24 in the IAPP fibril structures on a Ramachandran Plot. The favored areas in the Ramachandran plot are displayed in orange and allowed areas are displayed in yellow. If the structure contains symmetric chains the average values are plotted and if the structure contains asymmetric chains the two values are reported separately as Chain A and Chain B FIGURE S2: Triplicates of the thioflavin‐T kinetic assays. Details are provided in the captions to Figure‐3, Figure‐4 and Figure‐5 in the main manuscript. Figures 3, 4 and 5 each display one representative curve for each polypeptide. FIGURE S3: Results of seeding experiments (A) amyloid formation kinetics of hIAPP (Black), hIAPP seeded with pre‐formed hIAPP amyloid fibrils (Gray) and hIAPP seeded with pre‐formed G24L‐hIAPP fibrils (green) and (B) amyloid formation kinetics of hIAPP (Black), hIAPP seeded with pre‐formed hIAPP amyloid fibrils (Gray) and hIAPP seeded with pre‐formed G24 2Abu‐hIAPP fibrils (yellow) measured using the fluorescence of thioflavin‐T in 10 mM phosphate buffered saline (140 mM KCl), pH 7.4 and in 25°C TABLE S1. Energy decomposition (Total, Side Chain and Backbone energies) of the Van der Waal's energy of the 24th residue calculated after minimizing the fibril structures of hIAPP, G24 2Abu‐hIAPP, G24L‐hIAPP and G24P‐hIAPP for 10000 steps in Amber 20. If the two stacks of a fibril structure are inequivalent energies for both stacks are listed. [file PRO-32-e4539-s001.docx]

Supporting Information for

On the Plasticity of Amyloid Formation: The Impact of Destabilizing Small to Large Substitutions on IAPP Amyloid Formation

Lakshan Manathunga^1,3^, **Rehana Akter^1^, Alexander Zhyvoloup^2^,** Carlos Simmerling^1,3^ and Daniel P. Raleigh^1-3,*^

^1^Deartment of Chemistry, Stony Brook University, Nicolls Road, Stony Brook, New York 11794, United States

^2^ Research Department of Structural and Molecular Biology, University College London, Gower Street, London WC1E 6BT, United Kingdom

^3^Laufer Center for Physical and Quantitative Biology, Stony Brook University, Stony Brook, New York 11794, United States

* Author to whom correspondence should be addressed:

DPR email: daniel.raleigh@stonybrook.edu or d.raleigh@ucl.ac.uk phone: (631) 632-


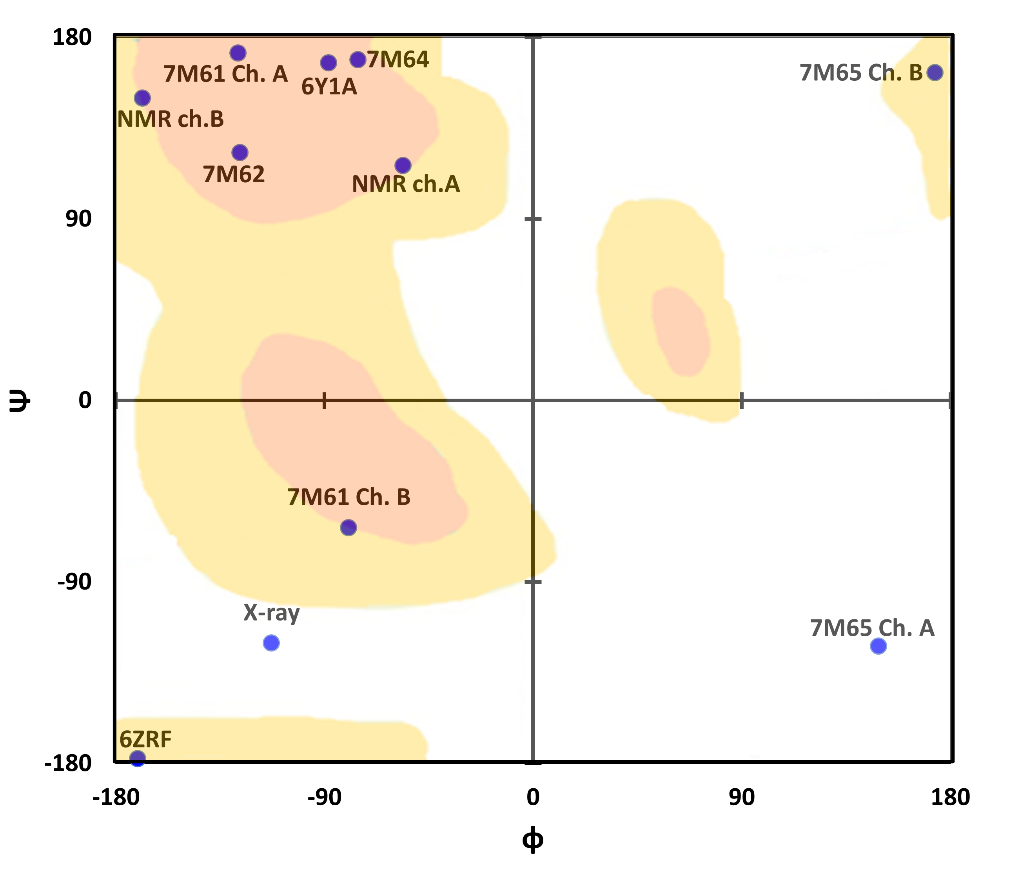


**Figure S1:** Placement of Φ, Ψ angles of Gly24 in the IAPP fibril structures on a Ramachandran Plot. The favored areas in the Ramachandran plot are displayed in orange and allowed areas are displayed in yellow. If the structure contains symmetric chains the average values are plotted and if the structure contains asymmetric chains the two values are reported separately as Chain A and Chain B.


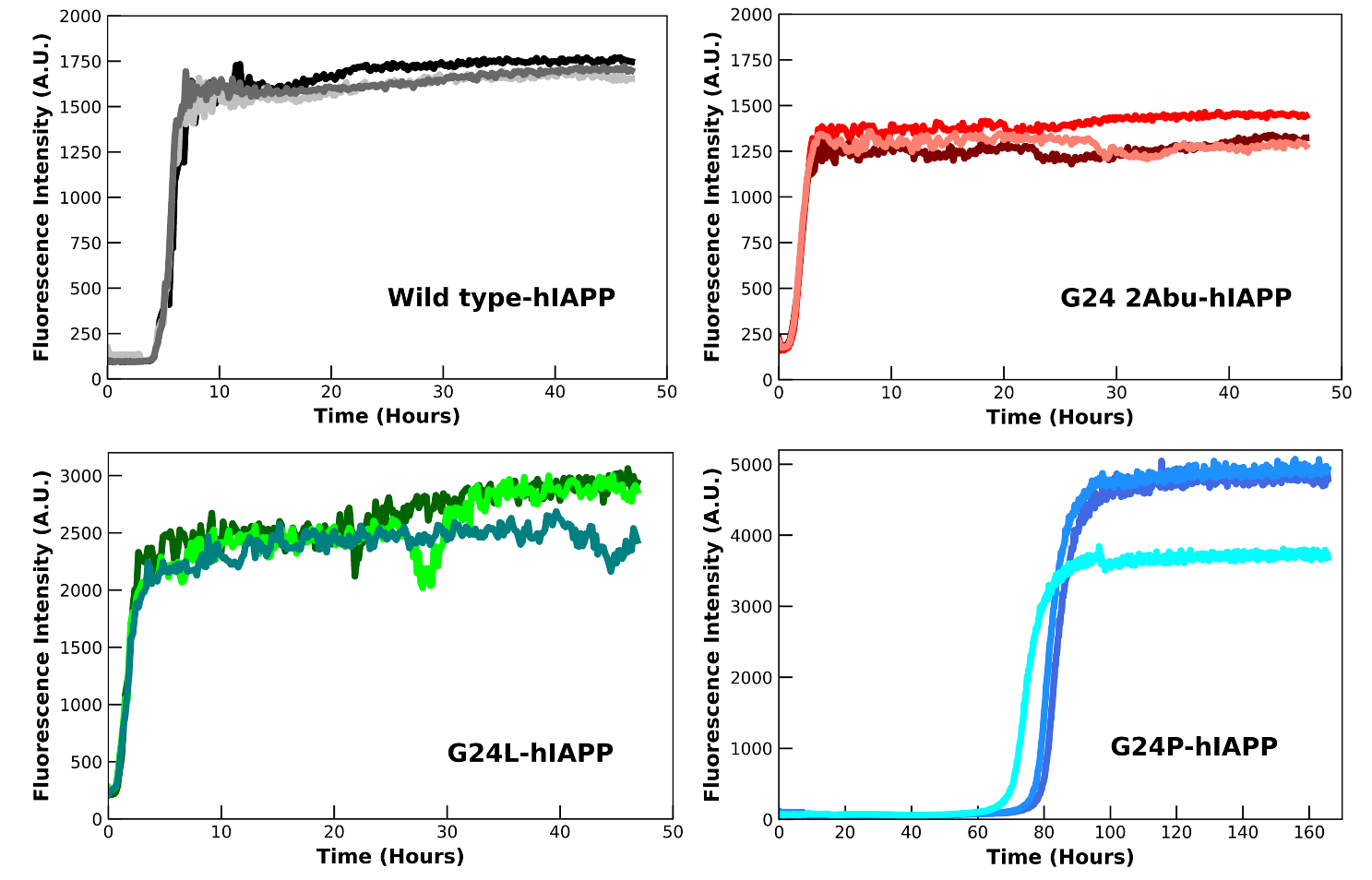


**Figure S2:** Triplicates of the thioflavin-T kinetic assays. Details are provided in the captions to Figure-3, Figure-4 and Figure-5 in the main manuscript. Figures 3, 4 and 5 displays one representative curve for each polypeptide.


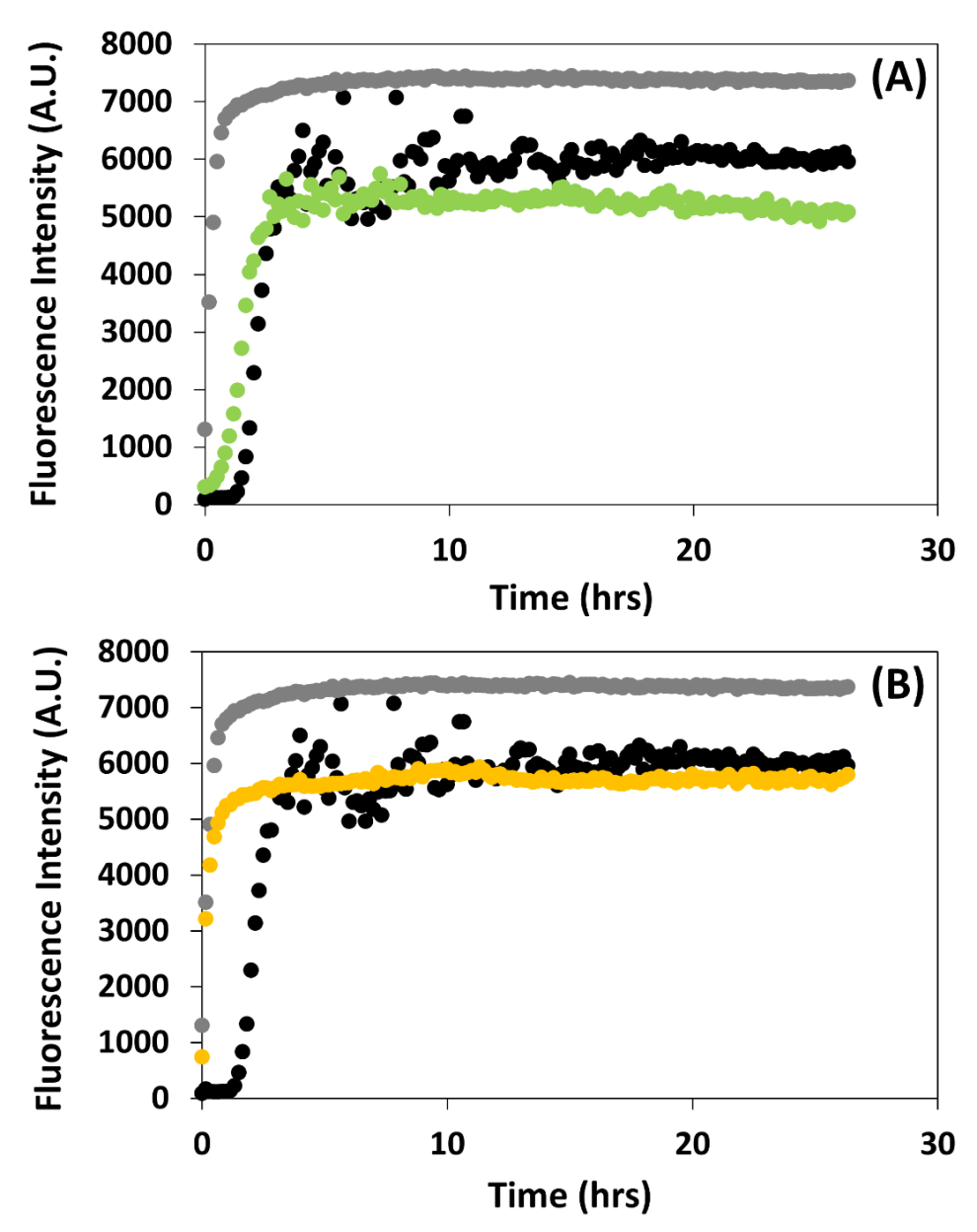


**Figure S3:** Results of seeding experiments **(A)** amyloid formation kinetics of hIAPP (Black), hIAPP seeded with pre-formed hIAPP amyloid fibrils (Grey) and hIAPP seeded with pre-formed G24L-hIAPP fibrils (green) and **(B)** amyloid formation kinetics of hIAPP (Black), hIAPP seeded with pre-formed hIAPP amyloid fibrils (Grey) and hIAPP seeded with pre-formed G24 2Abu-hIAPP fibrils (yellow) measured using the fluorescence of thioflavin-T in 10 mM phosphate buffer saline (140 mM KCl), pH 7.4 and in 25℃.

**Table-S1.** Energy decomposition (Total, Side Chain and Backbone energies) of the Van der Waal’s energy of the 24^th^ residue calculated after minimizing the fibril structures of hIAPP, G24 2Abu-hIAPP, G24L-hIAPP and G24P-hIAPP for 10000 steps in Amber 20. If the two stacks of a fibril structure are inequivalent energies for both stacks are listed.

| **PDB ID** | **Experimental Method** | **Van der Waal's Energy (Kcal mol^-1^)** | | | | | | | | | | | |
| --- | --- | --- | --- | --- | --- | --- | --- | --- | --- | --- | --- | --- | --- |
|  |  | **hIAPP** | | | **G24 2Abu - hIAPP** | | | **G24L - hIAPP** | | | **G24P-hIAPP** | | |
|  |  | **Total** | **Side Chain** | **Backbone** | **Total** | **Side Chain** | **Backbone** | **Total** | **Side Chain** | **Backbone** | **Total** | **Side Chain** | **Backbone** |
| (a) | x-ray | -1.4 | -1.1 | -0.3 | 38.1 | 29.8 | 8.3 | 23.9 | 17.7 | 6.2 | ***** | ***** | ***** |
| (a) - stack A | solid state NMR | -3.0 | -0.8 | -2.2 | 56.4 | 42.6 | 13.8 | ***** | ***** | 0.6 | -4.9 | -3.2 | -1.7 |
| (a) - stack B | solid state NMR | -2.7 | -0.5 | -2.2 | 304.1 | 239.9 | 64.2 | ***** | ***** | 0.0 | 2.6 | 2.0 | 0.5 |
| 6Y1A | Cryo-EM | -2.3 | -1.4 | -0.8 | 28.6 | 20.1 | 8.5 | ***** | ***** | ***** | 31.9 | 24.2 | 7.7 |
| 6ZRF | Cryo-EM | -2.0 | -0.9 | -1.1 | 17.6 | 13.4 | 4.2 | 44.9 | 32.0 | 12.9 | 25.9 | 17.2 | 8.6 |
| 7M61 - stack A | Cryo-EM | -3.3 | -1.3 | -2.1 | ***** | ***** | ***** | ***** | ***** | ***** | 55.7 | 33.8 | 21.9 |
| 7M61 - stack B | Cryo-EM | -3.9 | -0.9 | -3.0 | ***** | ***** | ***** | ***** | ***** | ***** | 33.4 | 24.0 | 9.4 |
| 7M62 | Cryo-EM | -3.0 | -1.1 | -1.9 | -1.8 | -1.4 | -0.4 | ***** | ***** | ***** | ***** | ***** | ***** |
| 7M64 | Cryo-EM | -3.1 | -1.1 | -2.0 | 34.6 | 25.9 | 8.7 | ***** | ***** | ***** | 33.3 | 26.1 | 7.2 |
| 7M65 - stack A | Cryo-EM | -2.1 | -0.5 | -1.6 | 16.1 | 13.7 | 2.4 | 115.9 | 78.1 | 37.7 | 35.9 | 27.9 | 8.1 |
| 7M65 - stack B | Cryo-EM | -1.7 | -1.0 | -0.6 | -1.0 | -0.8 | -0.2 | 6.1 | 5.0 | 1.1 | 55.5 | 41.3 | 14.2 |

(a) – Coordinates supplied by the authors

***** - High energy (greater than 999999.999 Kcal mol^-1^)
